# The Silent Majority: Aging With HIV

**DOI:** 10.1093/geroni/igab046.844

**Published:** 2021-12-17

**Authors:** Paul Nash, Molly Perkins

## Abstract

Those over the age of 50 represent the majority of people living with HIV (PWH), most of the HIV research, prevention and service retention work is targeted at ‘at-risk’ communities under age 50. Given this diverse and growing population, intersections of age with HIV need to be prioritized. This focus would actively increase quality of care and life experience for older PWH and the growing numbers transitioning into old age. Using local, national, and international data, this symposium will highlight the unmet social needs of older PWH. Presentations will provide evidence of unmet need, decreased self-esteem, enhanced health burden, and the damaging nature of stigma. Given the impact of COVID-19 globally, the data will further demonstrate the need to support immunocompromised older PWH. Older PWH are a marginalized community and the effects of COVID-19 have been disproportionately severe. With the adverse health outcomes experienced because of COVID-19 and intersectional stigma, it is important to understand the support structures that are and are not in place for older PWH. Advance care directives make up an integral part of future planning, especially for those living with chronic health concerns, yet little research has previously evidenced the steps taken by OPWH. Finally, using data from sub-Saharan Africa, emotional and instrumental social support sufficiency will be described to highlight the unmet needs of these older PWH. Our discussion will focus on the need for policies and programs to support this growing segment of the HIV population with increasingly diverse and unmet needs.

